# The effect of birth order on length of hospitalization for pediatric traumatic brain injury: an analysis of the 1987 Finnish birth cohort

**DOI:** 10.1186/s13690-022-00919-x

**Published:** 2022-07-11

**Authors:** Mazin Omer, Jussi P. Posti, Mika Gissler, Marko Merikukka, Ildiko Hoffmann, Till Bärnighausen, Michael Lowery Wilson

**Affiliations:** 1 Heidelberg Institute of Global Health (HIGH), University of Heidelberg, Im Neuenheimer Feld 130/3, 69120 Heidelberg, Germany; 2 Injury Epidemiology and Prevention (IEP) Research Group, Turku Brain Injury Center, Turku University Hospital and University of Turku, Turku, Finland; 3 Neurocenter, Department of Neurosurgery and Turku Brain Injury Center, Turku University Hospital and University of Turku, Turku, Finland; 4 Department of Knowledge Brokers, Finnish Institute for Health and Welfare (THL), Helsinki, Finland; 5 Region Stockholm, Academic Primary Health Care Centre, Stockholm, Sweden; 6 Department of Molecular Medicine and Surgery, Karolinska Institutet, Stockholm, Sweden; 7 Children, Adolescents and families, Finnish Institute for Health and Welfare (THL), Oulu, Finland; 8 Itla Children’s Foundation (Itla), Helsinki, Finland

**Keywords:** Traumatic brain injury, Birth order, Children, Pediatric, Hospital stay

## Abstract

**Purpose:**

This study examines the relationship between birth order and length of hospitalization due to pediatric traumatic brain injury (TBI).

**Methods:**

We prospectively followed 59,469 Finnish newborns from 1987 until age 18 years. Data on first diagnosis of TBI was recorded within the 1987 Finnish Birth Cohort (FBC). Hospitalization period was divided into two categories: 2 days or less and more than 2 days. The latter was considered in this study as longer hospitalization.

**Results:**

Compared with first born siblings, later born siblings had an increased risk of a longer hospitalization for TBI (12.7% of fourth or higher born birth children diagnosed with TBI were hospitalized for 2 or more days, 11.3% of first born, 10.4% of third born and 9.0% of second born). Fourth or higher born children were more likely to experience a repeat TBI; 13.4% of fourth or higher born children diagnosed with TBI had 2–3 TBIs during the study period compared to 9% of third born, 7.8% of second born and 8.8% of the first born. Injuries in the traffic environment and falls were the most common contributors to pediatric TBI and occurred most frequently in the fourth or higher birth category; 29.3% of TBIs among fourth or higher birth order were due to transport accidents and 21% were due to falls.

**Conclusions:**

This study revealed a significant increase in risk for longer hospitalization due to TBI among later born children within the same sibling group. The study provides epidemiological evidence on birth order as it relates to TBI, and its potential to help to explain some of the statistical variability in pediatric TBI hospitalization over time in this population.

**Supplementary Information:**

The online version contains supplementary material available at 10.1186/s13690-022-00919-x.

## Introduction

Traumatic brain injury (TBI) is one of the most common causes of disability and mortality among children and young adults worldwide [1]. Due to their physiological and anatomical differences, as well as the relatively difficult neurological evaluation compared to adults, children who are diagnosed with a TBI may have more serious short- and long-term consequences even with mild TBI. These consequences may include cognitive, psychological, social and physical sequalae [2–4]. Hence, pediatric TBI is considered a problem of significant clinical and public health importance [5]. In Finland, the annual incidence of TBI among children is 99/100,000 with a peak incidence occurring at the age of 6–7 years in both sexes [6].

Length of hospitalization (LOS) is considered an important marker to assess the effect and consequences of pediatric TBI. LOS after a pediatric TBI has been used a measure for costs, resource utilization and the quality of the service in health facilities [7]. Inpatient care for a child with severe TBI costs on average over USD 70,000 per episode [8] and between USD 1000–7000 in the first 3 months after injury for post hospital care and rehabilitation in the United States [8–10]. LOS has also implications for families who must often provide post-hospital care and bear the costs associated with treatment [8, 10, 11]. Hence, reducing LOS after pediatric TBI, whenever possible, is a main goal to health facilities, as it is associated with decrease hospital acquired infections and thus morbidity, mortality and costs for healthcare providers and families [10]. Therefore, understanding the risk factors that play a role in the LOS after pediatric TBI is of an importance. Some of the factors examined in the literature include demographic non-modifiable variables, such as gender, race and ethnicity [11]. The specific mechanisms of injury including falls, the severity of the injury and the accompanying initial trauma score are significantly associated with LOS [12, 13]. Furthermore, surgical operations, complications and post-trauma pain could also influence the LOS after a TBI [7, 14, 15].

Examining risks for TBI as well as underlying susceptibility for TBI has long been a subject of research interest [13]. It has been postulated that the childbirth position among siblings affects their psychosocial development [16]. Various factors including personal, genetic, and environmental as well as familial factors play an important role in formulating the relationship between birth order and health phenomena. In the literature, some studies had examined the relationship between birth order, various physical injuries and psychological conditions like depression and suicide attempts [17, 18]. Multiple studies suggest that the birth order of sibling order contribute to the risk of TBI, where late born children are more predispose to TBI in relation to their early born siblings [13, 17]. Nevertheless, the relation between TBI, birth order, and TBI-related LOS is not yet fully examined and understood. Investigation of such an association, which would also reflect other indirect risk factors including severity of TBI according to birth order and socioeconomic factors, would help to understand the impact of pediatric TBI on health care providers and families and thus to prevent and reduce it.

Thus, in the present study, we examine the association between later-born children and longer LOS due to TBI compared with to their early-born siblings. We also examine the association between birth order and repeated TBI and the most common contextual factors contributing to hospitalization of children with TBI in the Finnish population.

## Methods

### Study population

We performed a retrospective cohort study based on data derived from the 1987 Finnish Birth Cohort (FBC). The FBC is a longitudinal population based and nation-wide register, which includes a complete census of all children born in 1987 in Finland (*n* = 60,069). The FBC contains information on health and demographic circumstances from the perinatal period up to and including early adulthood (median follow up period: 12.2, interquartile range IQR: 7.5–15.7).

The cohort contains information pertaining to all live born infants and stillborn infants weighing more than 500 g or those with a gestational age of at least 22 weeks born in Finland in 1987. Detailed information about the cohort may be obtained elsewhere [19, 20]. The 1987 FBC has an ethics approval from the Finnish Institute for Health and Welfare (THL) (decision §28/2009) and all relevant permissions from all register keeping authorities. The legal basis for the processing of personal data is public interest and scientific research (EU General Data Protection Regulation 2016/679 (GDPR), Article 6(1)(e) and Article 9(2)(j); Data Protection Act, Sections 4 and 6). Since neither cohort members nor their guardians were contacted during the conduct of this study, no informed consent was required.

### Inclusion criteria and definitions

TBI diagnoses were coded according to the International Statistical Classification of Diseases and Related Health Problems (ICD-9: 1987–1995; ICD-10: 1996–2005) as follows: We operationally defined TBI to include cases of concussion, diffuse injury, focal intraparenchymal lesions (such contusions and intracerebral hematomas) and convexity hematomas, cranial nerve injuries and crush injuries as a result of trauma. Moreover, also fractures of the calvarium and skull base were included (ICD-9 codes 800–801, 803, 804 except for facial traumas, 850–854 and 950–951) (ICD-10 codes S06.0–S06.9, S02.0, S02.1, S02.7–S02.9, S04.0–S04.9, S07.1, S07.8, S07.9, S09.7–S09.9, T02.0, T04.0, T06.0). Cohort members who had been diagnosed with TBI in a hospital setting or visited outpatient clinics (since 1998) in the region were included in this series. Causes of TBI as well as co-morbidities at the time of the diagnosis of TBI were identified by using ICD-9 and ICD-10 as shown in Additional file 1: Annex 1 and Annex 2. Hospital diagnoses of TBI were obtained from the 1987 FBC which is based on linked and validated administrative register data [19].

For this study, we identified the children belonging to each set of parents by utilizing the combined parent birth dates that were registered in the Central Population Register for each child in the cohort. In order to reduce bias due to e.g., household disruption due to divorce, we restricted the analysis to intact family units consisting of both biological parents who had never been separated/divorced during the cohort period. This was done to reduce the probability of introducing factors such as having multiple single mothers with the same birth date, or different children residing concurrently with multiple or non-biological parents. We included all children with complete information who were followed up from birth until age 18 years (1987–2005), unless they died prior to the year at entry. The final number of included children in the study was 59,469. The data are presented in three periods (1987–1992, 1993–1998; 1999–2005).

### Statistical analysis

Because all children entered the observation window at the same time, 1987, age at entry reflects individual year of birth year and used as a control variable along with sex and mother’s age at delivery. All TBIs for cohort members were analyzed and calculated per 100,000 child-years. The follow-up ended, when the cohort member died or emigrated from Finland. Frequencies and percentages were used to present the data. The analyses were performed using RStudio Desktop 1.2.5042 for Microsoft Windows.

## Results

Between 1987 and 2005, 59,469 children (30,435 males and 29,041 females) were followed. Of them, 1614 (2.7%) were diagnosed with a TBI, of whom 61% (984) were boys and 39% (630) were girls. In total, 1445 (89.5%) were admitted to hospital for 2 days or less and 169 (10.4%) were admitted more than 2 days during the study period. As shown in Table 1 first born children had the highest admission due to TBI “for two days or less” and “more than two days” during the study period followed by second born then third born children and finally fourth or higher born children.

**Table 1:** Characteristics of Sibling Groups, 1987–2005

Variable	Hospital stay, 2 days or less ***n*** = 1445		Hospital stay, more than 2 days ***n*** = 169		Total	
	Frequency	%	Frequency	%	Frequency	%
**Birth order**						
First	544	37.6	69	40.8	613	38.0
Second	515	35.6	51	30.2	566	35.1
Third	249	17.2	29	17.2	278	17.2
Fourth or higher	137	9.5	20	11.8	157	9.7
**Age, Mean (SD)**	11.7 (4.97)		9.2 (4.90)		11.4 (5.01)	
**Sex**						
Male	875	60.6	109	64.5	984	61.0
Female	570	39.4	60	35.5	630	39.0
**years**						
1987–1992	225	15.6	39	23.1	264	16.4
1993–1998	388	26.9	76	45.0	464	28.7
1999–2005	832	57.6	54	32.0	886	54.9

As shown in Table 1, the most TBIs during the study were diagnosed between 1999 and 2005 followed by the period between 1993 and 1998 and finally between 1987 and 1992. Most of the children who stayed two or less days in a hospital were admitted in the time period between 1999 and 2005, followed by the period between 1993 and 1998 and finally between 1987 and 1992.

As shown in Table 2, when comparing the admissions due to TBI within each birth order category, a fourth or higher born child had the highest probability of being admitted more than 2 days due to a TBI followed by first born child, then third born child and finally second born child. Nevertheless, more than two-day admissions due to TBI declined over time among all the birth categories during the study period.

**Table 2:** Hospital admission due to isolated TBI among siblings according to birth order

	1987–1992		1993–1998		1999–2005		1987–2005	
Birth order	≤ 2 days Frequency (%)	> 2 days Frequency (%)	≤ 2 days Frequency (%)	> 2 days Frequency (%)	≤ 2 days Frequency (%)	> 2 days Frequency (%)	≤ 2 days Frequency (%)	> 2 days Frequency (%)
**First**	70 (85.4%)	12 (14.6%)	140 (81.4%)	32 (18.6%)	334 (93.0%)	25 (7.0%)	544 (88.7%)	69 (11.3%)
**Second**	92 (88.5%)	12 (11.5%)	147 (87.5%)	21 (12.5%)	276 (93.9%)	18 (6.1%)	515 (91.0%)	51 (9.0%)
**Third**	39 (83.0%)	8 (17.0%)	63 (81.8%)	14 (18.2%)	147 (95.5%)	7 (4.5%)	249 (89.6%)	29 (10.4%)
**Fourth or higher**	24 (77.4%)	7 (22.6%)	38 (80.9%)	9 (19.1%)	75 (94.9%)	4 (5.1%)	137 (87.3%)	20 (12.7%)

The incidence of TBI per 100,000 child-years (95% CIs) in the cohort for the whole study period (1987–2005) was as follows; 143.8 (132.4–155.2) for the earliest born, 146.4 (134.4–158.5) for the second born, 157.2 (138.8–175.8) for the third born, 183.8 (155.0–212.6) for the latest born, respectively (Table 3). Consistently, when comparing the incidence between the two categories (Hospital stay 2 days or less vs Hospital stay more than 2 days) for the whole study period (1987–2005), the incidence of diagnosed TBIs increased by birth order; (127.85 vs 16.44), (133.43 vs 13.39), (141.07 vs 16.67), (160.73 vs 23.86) for the earliest, second, third and latest born siblings, respectively.

**Table 3:** Incidence of TBI across birth order categories according to the period of hospital stay (2 days or less vs more than 2 days), 1987–2005

Hospital stay 2 days or less					Hospital stay more than 2 days				Overall			
**1987–1992** **Birth order**	**I**	**SE**	**LCI**	**UCI**	**I**	**SE**	**LCI**	**UCI**	**I**	**SE**	**LCI**	**UCI**
**1**	54.56	6.52	41.78	67.34	9.53	2.75	4.14	14.93	63.74	7.03	49.95	77.54
**2**	79.03	8.24	62.88	95.18	10.52	3.03	4.56	16.47	89.15	8.74	72.01	106.28
**3**	73.35	11.74	50.33	96.37	15.37	5.43	4.72	26.02	88.16	12.86	62.96	113.37
**4+**	93.40	19.06	56.03	130.77	27.89	10.54	7.22	48.55	120.20	21.58	77.89	162.52
**1993–1998** **Birth order**	**I**	**SE**	**LCI**	**UCI**	**I**	**SE**	**LCI**	**UCI**	**I**	**SE**	**LCI**	**UCI**
**1**	52.39	4.42	43.71	61.07	12.21	2.15	7.98	16.44	64.20	4.89	54.61	73.80
**2**	60.58	4.99	50.79	70.38	8.84	1.92	5.05	12.62	69.12	5.33	58.66	79.57
**3**	56.82	7.15	42.78	70.85	12.90	3.44	6.14	19.66	69.26	7.89	53.79	84.74
**4+**	70.84	11.49	48.31	93.36	17.19	5.73	5.96	28.42	87.29	12.73	62.34	112.25
**1999–2005** **Birth order**	**I**	**SE**	**LCI**	**UCI**	**I**	**SE**	**LCI**	**UCI**	**I**	**SE**	**LCI**	**UCI**
**1**	78.06	4.27	69.69	86.44	5.95	1.19	3.61	8.28	83.68	4.41	75.02	92.33
**2**	70.99	4.27	62.61	79.36	4.72	1.11	2.54	6.90	75.45	4.40	66.82	84.07
**3**	82.72	6.82	69.35	96.10	4.01	1.51	1.04	6.99	86.41	6.96	72.76	100.06
**4+**	87.24	10.07	67.50	106.99	4.76	2.38	0.09	9.42	91.52	10.29	71.34	111.71
**1987–2005** **Birth order**	**I**	**SE**	**LCI**	**UCI**	**I**	**SE**	**LCI**	**UCI**	**I**	**SE**	**LCI**	**UCI**
**1**	127.85	5.48	117.11	138.60	16.44	1.97	12.56	20.32	143.84	5.80	132.45	155.23
**2**	133.43	5.87	121.91	144.96	13.39	1.87	9.72	17.07	146.46	6.15	134.40	158.53
**3**	141.07	8.94	123.55	158.60	16.67	3.09	10.60	22.74	157.29	9.43	138.80	175.78
**4+**	160.73	13.73	133.81	187.64	23.86	5.33	13.40	34.31	183.85	14.67	155.09	212.61

*I* Incidence, *SE* Standard error of I, *LCI* Lower bound of confidence interval (95%CI), *UCI* Upper bound of confidence interval (95%CI)

Concerning repeated TBI, the number of children who had been diagnosed with 4 or more TBIs during the study period, slightly increased within all the birth categories overtime as shown in Table 4. Fourth or higher born children had the highest percentage of 2–3 TBI study period (1987–2005); 21 (13.4%) of TBIs among fourth or higher born children in comparison to 25 (9%) among third born children, 44 (7.8%) among second born and 54 (8.8%) among first born children.

**Table 4:** Number of repeated hospital admission among children with the discharge diagnosis of TBI according to birth order

	1987–1992*			1993–1998*			1999–2005*			1987–2005*		
Birth order	One TBI	2–3 TBIs	4 TBIs or more	One TBI	2–3 TBIs	4 TBIs or more	One TBI	2–3 TBIs	4 TBIs or more	One TBI	2–3 TBIs	4 TBIs or more
**First**	72 (93.5%)	5 (6.5%)	0	155 (88.6%)	9 (5.2%)	11 (6.2%)	298 (82.6%)	40 (11.1%)	23 (6.3%)	525 (86.6%)	54 (8.8%)	34 (5.5%)
**Second**	94 (87%)	5 (4.6%)	9 (8.4%)	155 (94.5%)	9 (5.6%)	0	250 (85.0%)	30 (10.3%)	14 (4.7%)	499 (88.2%)	44 (7.8%)	23 (4.1%)
**Third**	43 (93.4%)	3 (6.6%)	0	70 (89.7%)	5 (6.5%)	3 (3.8%)	126 (81.8%)	17 (11.1%)	11 (7.1%)	239 (86.0%)	25 (9.0%)	14 (5.0%)
**Fourth or higher**	29 (87.8%)	3 (9.1%)	1 (3.1%)	39 (82.9%)	8 (17.1%)	0	61 (79.3%)	10 (12.9%)	6 (7.8%)	129 (82.2%)	21 (13.4%)	7 (4.5%)

Transport accidents followed by falls were the most common contributors to TBIs among cohort members (Table 5). A total of 29.3% of TBIs among fourth or higher birth order children were due to collisions in the traffic environment followed by 28.3% among second born children, 27.6% among first born children and 24.8% among third born children. Consistently, 21% of TBIs among fourth or higher birth order were due to falls. Almost 50% of the diagnosed cases of TBI in all birth categories had an unknown etiology.

**Table 5:** External causes of hospital admission due to TBI among children 1987–2005

	Falls		Traffic collisions		Violence		Others		Unknown		Total	
Birth order	Frequency	%	Frequency	%	Frequency	%	Frequency	%	Frequency	%	Frequency	%
First (*n* = 613)	99	16.2	169	27.6	11	1.8	13	2.1	321	52.4	613	100
Second (*n* = 566)	88	15.5	160	28.3	4	0.7	15	2.7	299	52.8	566	100
Third (*n* = 278)	45	16.2	69	24.8	4	1.4	5	1.8	155	55.8	278	100
Fourth or higher (*n* = 46)	33	21.0	46	29.3	1	0.6	2	1.3	75	47.8	157	100

## Discussion

This study examined the relationship between sibling birth order and LOS due to TBI. It also examined the relationship between birth order and repeated TBI, as well as the most common causes of pediatric TBI.

The study found that a higher risk of TBI was associated with fourth or higher birth order and a higher likelihood of having been hospitalized for two or more days.

Hospitalization period were divided into two categories; 2 days or less and more than 2 days, as most of the discharges of mild cases occurs within 1–2 days.

Severity of the initial injury has been shown to be one of the most important factors influencing LOS [21] with patients with moderate to severe TBIs tending have longer LOS. This study found that half of TBI cases occurred in the road traffic environment or were the result of falls, which is consistent with the existing literature [22–25]. The highest rates of both traffic- and fall-related TBIs occurred in those born fourth or higher order.

Relevant comorbidities that could influence the LOS of TBI patients are neurological and psychological disorders, which were rare (only 7 children) in our study and therefore did not affect the in LOS due to TBI. Risk factors for a prolonged recovery period have included age, with some studies showing that very young children and adolescents require more time to recover from mild TBI [26, 27]. Although TBI is more common among males as shown in this study and in the literature [28, 29] due to their involvement in rough competitive play, aggressiveness and social expectations, which accept aggression among boys [30–32], being a female has been shown to be a risk factor for a prolonged recovery [33]. Moreover, recent studies shown that having a history of repetitive concussions leads to more significant physical and cognitive consequences, prolonged recovery and LOS [33–37]. American athletes repeatedly exposed to mild TBIs have been found to have increased rates of cognitive impairment and long-term psychiatric illness [37]. In the present study, firstborns, and children of fourth or higher birth order had a higher prevalence of repeated TBIs.

Delayed hospital discharge might further be caused by factors related to the domestic situation. A large family, a noisy environment at home, parental concerns or the suspicion of domestic violence may lead physicians to delay hospital discharge [38]. Apart from that later-born siblings have been considered riskier in their behavior compared to their younger siblings due to differences in parental supervision [39], the size of the household may also have an influence on parental supervision [40]. Birth order category may be a valuable parameter in risk assessment of pediatric TBI patients. Researchers found that later-born siblings were more likely to be hospitalized for injuries, which could be related to less parental attention [41]. Buur et al. showed in their data that living in a household with 4 or more children increases the risk of sustaining injuries, which results in hospitalization among siblings [42]. This would explain as well as the higher incidence of repeated TBI among later born children. This situation in the home environment could lead to a delayed hospital discharge to prevent a prolonged recovery period. Delayed recovery period may lead to negative long-term consequences such as reduced intellectual performance as well as psychiatric disorders and premature mortality [43], so undisturbed and fast recovery should be a therapeutic goal.

Moreover, our study found that the prevalence of TBI increased, and LOS decreased in all birth categories, as reflected by the increase in the admission rate of less than 2 days over time. This is consistent with the literature, in which the average incidence of TBI increases over time globally, as shown by data from two systemic reviews by Tagliaferri et al. for years 1980–2003 [44] and 1990–2014 [45]. In this regard, the improvement in the diagnostic capabilities and their availability could lead to detection of more TBI cases [46]. Nevertheless, mild TBI accounted for 70–90% of all diagnosed TBIs [47]. The increase in diagnosed cases of mild TBIs over time has also been attributed also to the increasing availability of new diagnostic and imaging technology [48].

One of the strengths of this study is the large, fully enumerated population cohort and the long follow-up period. The data were collected through a national health system, which assures uniform access to health care and hence a homogeneous, standardized data collection process. One of the limitations of the study which was not considered is multiple births, in which the age ranges are thus similar, exposing the entire sibling group to similar risk profiles as they develop. Another limitation of the study is that injury severity was not available as the hospital discharge register does not provide Glasgow Coma Scale (GCS) data, which would help in the examination of the effect of severity on LOS according to birth order. Data on extra-cranial injuries could not be included in the study, which could affect as well the LOS. There is also an aspect in the inclusion criteria that requires attention. We have included patients with calvarial and skull base fractures. The rationale for this is that possible forces, strong enough to cause a skull fracture may injure the underlying brain or result in intracranial bleeding such epidural hematoma.

## Conclusion

We demonstrate an association between fourth or higher in birth order sequence and length of hospital stay due to pediatric TBI. More research is needed in order to examine the relationship between birth order and TBI. Such studies may include additional analyses where GCS data are available in addition to potentially cross-national studies which might validate the findings of the present study. This study provides epidemiological evidence on birth order as it relates to TBI, and its potential to help to explain some of the statistical variability in pediatric TBI risk over time in this population.

Of general importance in reducing pediatric TBIs, regardless of birth order, are passive and active public health-strategies in the home environment. Such measures include the use of physical barriers such as safety gates erected at the tops of stairs, anti-slip surfaces, the use of night-lights, safety straps on chairs and active supervision of infants and toddlers on high places such as beds and sofa chairs. Furthermore, other preventive measures include the use of helmets as well as obeying traffic rules and signals while biking and skating. Playing sports in a responsible manner by learning e.g. proper heads-up tackling techniques and avoid helmet-to-helmet contact. To avoid driving cars while Texting or under the influence of alcohol. The education of parents around fall prevention strategies as well as the correct use of car seats in vehicles are also of paramount importance.

## Supplementary Information

**Additional file 1: Annex 1.** Co-morbidities; Psychological and neurological disorders at the time of the diagnosis of TBI. **Annex 2.** Causes of Traumatic brain injury.

## Data Availability

The data that support the findings of this study are not publicly available due to EU data privacy agreements but are available from THL Finnish Institute for Health and Welfare upon reasonable request (info@thl.fi).
