# Supplementary material for: The effect of birth order on length of hospitalization for pediatric traumatic brain injury: an analysis of the 1987 Finnish birth cohort
Source: Arch Public Health. 2022 Jul 11;80:167. doi: 10.1186/s13690-022-00919-x (PMC9275049; doi:10.1186/s13690-022-00919-x)
Supplement: Supplementary file 1 — Additional file 1: Annex 1. Co-morbidities; Psychological and neurological disorders at the time of the diagnosis of TBI. Annex 2. Causes of Traumatic brain injury. [file 13690_2022_919_MOESM1_ESM.docx]

**Annexes:**

**Annex 1:**

Co-morbidities; Psychological and neurological disorders at the time of the diagnosis of TBI:

| **Diagnosis** | **ICD-10**^a^ | **ICD-9** |
| --- | --- | --- |
| Behavioral and emotional disorders  with onset usually occurring in  childhood and adolescence | (F90-F98) | (300-316)- 299- 783.6- V69.1- 296 – 293.83 - 297 – 295 |
| Neurotic, stress-related and somatoform disorders | (F40-F48) |  |
| Unspecified mental disorder | F99 |  |
| Disorder of psychological development | F80-F89 |  |
| Eating disorders | F50-F59 |  |
| Schizophrenia, schizotypal and delusional disorder | (F20-F29) |  |
| Intellectual disabilities | (F70-F79) |  |
| Extrapyramidal and movement disorders | (G20-G26) | (350-359)- 333- 728- 343- 344- 438.5 - 742.9 – 225- 228.02 – 237.7 – (215.0-215.9) - 192.1 – 225.2 – 742- 345 - 333.2 – 331- V17.1 |
| Episodic and paroxysmal disorders | (G40-G47) |  |
| Nerve, nerve root and plexus disorders | (G50-G59) |  |
| Polyneuropathies and other disorders of the peripheral nervous system | (G60-G64) |  |
| Diseases of myoneural junction and muscle (G70-73) | (G70-G73) |  |
| Cerebral palsy and other paralytic syndromes (G80-83) | (G80-G83) |  |
| Other disorders of the nervous system (G90-99) | (G90-G99) |  |
| Benign neoplasms of brain and other parts of central nervous system | D33 |  |
| Congenital malformations of nervous system | (Q00-Q07) |  |
| Cerebrovascular accident | I64 |  |
| Malignant Neoplasm of Brain | C71 |  |
| Malignant Neoplasm of Spinal cord, cranial nerves and other parts of central nervous system | C72 |  |
| Benign Neoplasm of Meninges | D32 |  |
| Sequelae of Intracranial Injury | T90.5 |  |

a International Statistical Classification of Diseases and Related Health Problems (ICD-9: 1987–1995; ICD-10: 1996–2005)

**Annex 2:**

Causes of Traumatic brain injury

|  | **ICD-10**^a^ | **ICD-9** |
| --- | --- | --- |
| **Traffic** | (V01–V89) | (E800–E807)  (E810–E819)  (E826–E829)  (E846–E848) |
| **Falls** | (W00-W19) | (E880–E888) |
| **Violence** | (X85-Y09) | (E960–E969) |
| **Other external causes** | (X30-X39)  (X58-X59)  (X60-X84) | (E900–E909)  (E916–E928)  (E950–E959) |

a International Statistical Classification of Diseases and Related Health Problems (ICD-9: 1987–1995; ICD-10: 1996–2005)
